# Cortical thickness analysis combined with CSF dynamics improves diagnostic stratification in idiopathic normal pressure hydrocephalus

**DOI:** 10.1007/s10143-026-04200-5

**Published:** 2026-03-10

**Authors:** Daniele Piccolo, Daniele Bagatto, Serena D’Agostini, Maria Cristina De Colle, Enrico Belgrado, Yan Tereshko, Marco Vindigni, Francesco Tuniz

**Affiliations:** 1 Department of Clinical, Diagnostic and Pediatric Sciences, University of Pavia, Via Alessandro Brambilla, 74, Pavia (PV), 27100 PV Italy; 2 Unit of Neurosurgery, Department of Head-Neck and Neuroscience, Azienda Sanitaria Universitaria Friuli Centrale, Udine (UD), 33100 Italy; 3 Unit of Neuroradiology, Department of Diagnostic Imaging, Azienda Sanitaria Universitaria Friuli Centrale, Udine (UD), 33100 Italy; 4 Unit of Neurology, Department of Neuroscience, Azienda Sanitaria Universitaria Friuli Centrale, Presidio Ospedaliero Universitario Santa Maria della Misericordia, Piazzale Santa Maria della Misericordia, 15, Udine, UD 33100 Italy

**Keywords:** Artificial intelligence, Cortical thickness, Normal pressure hydrocephalus

## Abstract

The diagnostic landscape for idiopathic normal-pressure hydrocephalus is intricate, and there is a pressing need for accurate and cost-effective methods. Because of the lack of accurate diagnostic and prognostic quantitative biomarkers, the frequent presence of comorbidities, and the limited understanding of the pathophysiology of the disorder, only a minority of patients receive disease-specific treatment. While traditional neuroimaging offers insights, its isolated diagnostic precision can be enhanced. Emerging quantitative methods analyzing cortical thickness based on standard T1-weighted brain MR images offer new diagnostic possibilities. We analyzed 294 patients referred to our clinic from January 2015 until December 2022. After the exclusion criteria, the final sample consisted of 100 possible iNPH patients. Of these, 71 underwent ventriculoperitoneal shunt surgery, while 29 did not qualify post-evaluation. Cortical thickness was assessed using an advanced deep-learning neuroimaging pipeline. For predictive modeling, we employed a comprehensive set of Machine Learning algorithms, including Distributed Random Forests, Extremely Randomized Trees, Generalized Linear Model with Regularization, Gradient Boosting Machines, Extreme Gradient Boosting machines, and a fully connected multi-layer Artificial Neural Network. These algorithms were strategically combined into a Super Learner ensemble approach to harness their collective predictive power. Among patients with negative CSFTT outcomes or subpar VPS surgery responses, distinct cortical variations emerged, particularly in the caudal middle frontal, rostral middle frontal, superior frontal, and superior parietal regions. Our Super Learner model, integrating CSF dynamics and cortical thickness data, achieved a 90% positive predictive value, signifying a tangible advancement over traditional measures. Analyzing preoperative cortical thickness emerges as a viable strategy for streamlining therapeutic decisions for potential iNPH patients. Future endeavors should focus on large-scale multicentric studies to further delineate specific cortical thickness patterns, potentially enhancing the prediction accuracy for VPS surgery outcomes.

## Background

Idiopathic normal-pressure hydrocephalus (iNPH) is a neurological condition associated with ventricles enlargement and unchanged cerebrospinal fluid (CSF) pressure, along with cognitive impairment, gait disturbance, and urinary dysfunction [1]. The prevalence of iNPH among adults aged 65 years or older is reported at 3.7%. Remarkably, this prevalence substantially increases to 8.9% in individuals aged 80 years and above [2].

While optimal treatment for idiopathic iNPH is reasonably known, diagnosis remains a topic of debate and scientific research [3]. No pathognomonic sign, laboratory results, or imaging findings are sufficient to establish a diagnosis of iNPH, which requires convergent evidence from clinical history, physical examination, diagnostic procedures, and brain imaging [4].

The most significant brain imaging finding in iNPH is ventriculomegaly [5, 6]. However, ventricular enlargement can also occur in neurodegenerative diseases and healthy elderly individuals [7]. Ventriculomegaly, dilation of the Sylvian fissure, and narrowing of the high parietal convexity or midline subarachnoid spaces define disproportionately enlarged subarachnoid-space hydrocephalus (DESH) [8]. DESH findings have high positive but low negative predictive values for iNPH [3]. In addition, some elderly individuals have findings similar to DESH on MRI, even though they are asymptomatic [9].

Other imaging methods rather than structural MRI are suitable for iNPH diagnoses, such as diffusion MRI [10–12], perfusion MRI [11], phase-contrast MRI [13], functional MRI [14], proton magnetic resonance spectroscopy [15], and nuclear medicine diagnostic methods [16, 17]. However, these diagnostic methods are still a current topic of debate in the literature, and their diagnostic value is still unproven [3].

Several studies on iNPH and CSF tap test (CSFTT) have demonstrated this diagnostic procedure’s high Positive Predictive Value (PPV) for successful shunt surgery [18]. However, shunt surgery is not consistently effective even with a positive CSFTT [18, 19, 20]. Instead, a negative CSFTT cannot reject the possibility that the symptoms will improve by a shunt intervention due to low negative predictive value and sensitivity [3, 18, 21–23].

iNPH symptoms overlap with other diseases, such as Alzheimer’s disease (AD), Parkinson’s spectrum (PS) disorder, and vascular dementia (VaD), which can also concurrently affect the patient, contributing to the symptomatology [24, 25]. Various tests, biomarkers, and neuroimaging techniques can be used to diagnose such diseases and comorbidities [3, 26]. However, their diagnostic accuracy is still suboptimal [3].

Quantitative computational methods for extracting cortical thickness measures have become available in the last decade as publicly available software packages. They only require T1-weighted brain MR images regularly available to diagnose a possible iNPH patient. Reportedly, distinctive cortical thinning patterns are associated with iNPH [27, 28, 29], AD [30–32], PS disorder [33], and VaD [34].

The use of Artificial Intelligence (AI) and Machine Learning (ML)-based solutions in surgery has shown potential in aiding diagnosis, predicting surgical outcomes, and optimizing surgical procedures. Recent studies have explored AI and ML applications in iNPH diagnosis and treatment prediction [35]. Sotoudeh et al. reported that using only clinical data, the Random Forest (RF) model achieved an area under the curve (AUC) of 0.71 and an accuracy of 0.70. In contrast, the Support Vector Machine (SVM) model, with the addition of Radiomics analysis, achieved an AUC of 0.80 and an accuracy of 0.76 [36]. Conversely, Mládek et al., using the lumbar infusion test, found eXtreme Gradient Boosting (XGB) to be the top-performing ML algorithm with an AUC of 0.891, outperforming traditional manual classification [37].

In this retrospective study, we analyzed the differences in cortical thickness among all possible iNPH patients referred to our clinic between January 2015 and December 2022 and evaluated its relationship with clinical assessments. Our hypothesis centered on identifying distinct cortical thinning patterns in patients with negative CSFTT outcomes and those unresponsive to shunt surgery, potentially elucidating underlying comorbidities. To achieve this, we employed multiple ML algorithms, including Distributed Random Forest (DRF), Extremely Randomized Trees (XRT), Generalized Linear Model (GLM) with Regularization, Gradient Boosting Machines (GBM), eXtreme Gradient Boosting machines (XGB), and a fully connected multi-layer Artificial Neural Network (Deep Learning machine). These algorithms were strategically integrated into a Super Learner ensemble approach to harness their collective predictive power [38].

## Methods

### Study populations

All patients referred to our clinic between January 2015 and December 2022, who met the diagnostic criteria for possible iNPH [3], underwent a comprehensive brain MRI and clinical assessment before CSFTT, at 24 h, and seven days post-CSFTT.

Patients presenting with CSF pressure below 200 mmH2O, standard CSF composition, and either the presence of neuroimaging indicators consistent with DESH along with gait disturbance or a clinical improvement following CSFTT were classified as probable iNPH [3] and were recommended for surgical intervention.

This study was approved by the local ethics committee (RIF. Prot IRB-DMED: 090/2021), and informed consent was obtained from each participant.

### Clinical evaluation

A total of 294 possible iNPH patients underwent neuropsychological, physiotherapeutic, and neurological examinations. The same neurologist assessed the patients before and after the test to eliminate inter-observer discrepancies. Motor and gait functions were evaluated using the Timed Up and Go test (TUG) and the Unified Parkinson’s Disease Rating Scale (UPDRS). An improvement of at least 10% in the TUG after CSF drainage was considered significant [3].

Cognitive function was evaluated using Mini-Mental State Examination (MMSE) and Frontal Assessment Battery (FAB). Urinary incontinence was documented as a binary variable (present/absent) based on clinical history and physical examination.

### CSF Dynamic evaluation

A Tuohy spinal needle was connected to a Möller Medical LiquoGuard 7 (Fulda, Germany) pressure monitor and fluid infusion system with the subject placed in the lateral recumbent position. After measuring baseline CSF pressure (P_start_), saline solution was infused at a constant rate of 1.5 mL/min until reaching a stable pressure plateau (P_plateau_). The resistance to outflow (R_out_) was calculated as the difference between P_plateau_ and P_start_ divided by the infusion rate, and a value higher than 12 mmHg/mL/min was considered significant.

After the infusion test, CSF was drained until the pressure returned to baseline. Then, a tap test was performed, draining up to 50 ml of CSF or until the pressure reached 0 mmH2O.

### MRI Data acquisition

Brain imaging was executed on a Philips Achieva 3 T whole-body scanner (Best, Netherlands) equipped with a SENSE-Head eight-channel head coil. The neuroradiological protocol included volumetric T1-weighted, T2-weighted, gradient-echo steady-state, phase contrast, diffusion-weighted, and fluid-attenuated inversion recovery (FLAIR) imaging. On these sequences, Evans’ index (EI), callosal angle (CA), and presence of Disproportionately Enlarged Subarachnoid-space Hydrocephalus (DESH) were recorded [3].

### MRI Data preprocessing

T1-weighted MRIs were processed with the Computational Analysis Toolbox (CAT, version 12.8.2) within SPM12 using MATLAB (version 2019a). All images were normalized using an affine followed by non-linear registration, corrected for bias field inhomogeneity, and followed by unified segmentation [39]. The DARTEL algorithm was used to spatially normalize the segmented scans into a standard MNI space. All segmented, modulated, and normalized GM and WM images were smoothed using 8-mm full-width-half-maximum Gaussian smoothing. Cortical thickness was measured according to the Desikan-Killiany-Tourville (DKT) atlas using the projection-based thickness method [40]. All data were visually inspected in each step for potential artifacts and inaccuracies in the surface reconstructions.

### Surgical technique

All probable-iNPH patients underwent ventricular-peritoneal shunt (VPS) implantation to the right ventricular frontal horn with an Integra LifeSciences CODMAN^®^ HAKIM^®^ Programmable Valve (Princeton, USA). All patients were discharged from the hospital within 4 days after VPS implantation and repeated the clinical evaluation one month, three months, six months, and one year later. There were no post-operative complications.

### Study groups

**Fig. 1:**
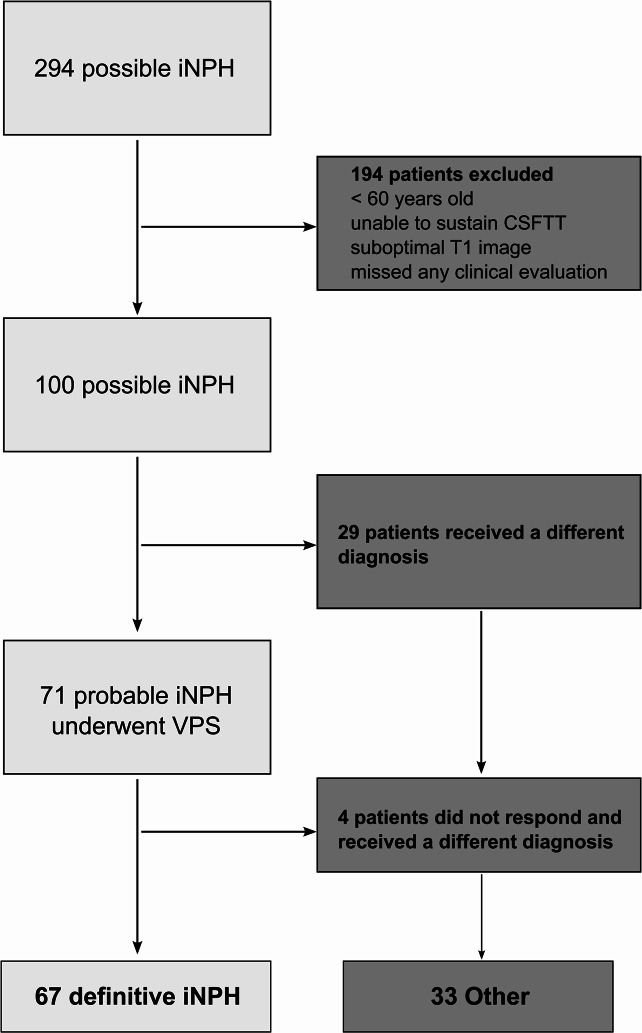
STROBE flow diagram of the study

Patients under 60 years old at the time of surgery, unable to sustain a CSFTT, missed any clinical evaluation or declined to participate were excluded from the study. The final population comprised 100 patients. Of these, 71 underwent CSF shunt surgery: 67 responded positively to the surgery and were thus confirmed as iNPH patients, while 4 did not show improvement and, following confirmation of a functional shunt, received alternative diagnoses. The remaining 29 were not indicated for surgery and ultimately received other diagnoses. For this study, the participants were categorized into two groups: iNPH, consisting of 67 patients, and ‘Other’, comprising 33 patients (Fig. 1).

Patients were classified as shunt-responsive or non-responsive based on a composite clinical evaluation incorporating all three components of the iNPH triad.

### Statistical analyses

Continuous variables were summarized as means with standard deviations (SD), while categorical variables were reported as frequencies and percentages. Group differences were assessed using effect size measures, including Cohen’s D and the standardized mean difference. The Wilcoxon rank-sum test was applied for non-normally distributed continuous variables, and Pearson’s Chi-squared test was used for categorical variables. Differences in cortical thickness between groups were evaluated using analysis of covariance (ANCOVA), with age and sex as covariates. To account for multiple comparisons, p-values were corrected using the Benjamini–Hochberg false discovery rate procedure. Pearson’s product-moment correlation coefficients were used to assess associations between clinical measures and MRI-derived metrics.

For predictive modeling, the dataset was stratified into a training set (70%) and an independent test set (30%), preserving the distribution of the outcome variable. All preprocessing steps, including Box–Cox transformation, centering, and scaling, were performed using parameters derived exclusively from the training data and applied within each cross-validation fold, ensuring that the test set remained completely unseen during model development.

To accommodate nonlinear relationships, feature interactions, and multicollinearity in a limited-sample setting, multiple machine learning algorithms were evaluated, including Distributed Random Forests (DRF), Extremely Randomized Trees (XRT), Generalized Linear Models with regularization (GLM), Gradient Boosting Machines (GBM), Extreme Gradient Boosting (XGB), and fully connected Deep Learning models. Model development and selection were conducted exclusively on the training set.

Ten-fold cross-validation was employed within the training set to compare predictor configurations, select the optimal algorithm for each configuration, and perform hyperparameter tuning, using the area under the receiver operating characteristic curve (AUC) as the primary performance metric. Following configuration selection, a stacked ensemble Super Learner was constructed to integrate the predictions of the best-performing base learners. Only after finalizing model architecture and hyperparameters was the selected Super Learner retrained on the full training set and evaluated once on the independent test set.

Feature importance and model interpretability were explored using SHapley Additive exPlanations (SHAP) for the leading tree-based model.

All statistical tests were two-tailed, with statistical significance defined as *p* < 0.05. Analyses were performed using RStudio (version 2023.06.1, Build 524).

## Results

Table 1 reports population characteristics, clinical assessments, CSF dynamic evaluation, and MRI metrics. There were no significant differences in the distribution of age and gender between the two groups.

**Table 1:** Demographics, clinical assessments, CSF dynamics, and MRI metrics

		Group			
Characteristic	**Overall**, *N* = 100^*1*^	**iNPH**, *N* = 67^*1*^	**Other**, *N* = 33^*1*^	Effect Size^2^	*p*-value^3^
**Demographics**					
Age	75.75 (5.34)	75.40 (4.60)	76.45 (6.61)	−0.20	0.2
Sex				0.11	0.6
F	57 (57%)	37 (55%)	20 (61%)		
M	43 (43%)	30 (45%)	13 (39%)		
**Clinical assessments**					
UPDRS					
before CSFTT	19.10 (12.42)	18.71 (13.69)	20.18 (16.95)	−0.12	0.7
after CSFTT	17.52 (12.53)	16.58 (10.41)	20.18 (17.54)	−0.29	> 0.9
difference	−1.57 (2.39)	−2.13 (2.47)	0.00 (1.18)	−0.96	**0.016**
TUG					
before CSFTT	19.54 (14.82)	18.71 (13.69)	21.86 (18.16)	−0.21	0.8
after CSFTT	18.63 (15.07)	16.98 (13.36)	23.27 (19.05)	−0.42	0.5
% variation	4.95 (14.49)	8.61 (11.83)	−5.36 (16.82)	1.10	**0.019**
MMSE					
before CSFTT	21.67 (4.90)	22.80 (4.13)	18.12 (5.69)	0.99	**0.031**
after CSFTT	22.64 (4.85)	24.04 (3.60)	18.25 (5.85)	1.30	**0.007**
difference	0.97 (1.05)	1.24 (1.05)	0.12 (0.35)	1.26	**0.005**
FAB					
before CSFTT	10.85 (3.25)	11.11 (3.18)	10.18 (3.49)	0.28	0.5
after CSFTT	11.59 (3.42)	12.07 (3.23)	10.36 (3.75)	0.50	0.2
difference	0.74 (1.04)	0.96 (1.10)	0.18 (0.60)	0.81	**0.019**
Incontinence	57 (57%)	39 (58%)	18 (54%)	0.08	0.5
**CSF dynamic evaluation**					
P start	9.88 (2.58)	10.11 (2.80)	9.42 (2.05)	0.27	0.2
P plateau	27.45 (4.71)	28.80 (4.19)	24.91 (4.63)	0.89	**< 0.001**
R out	11.43 (3.21)	12.02 (3.22)	10.26 (2.89)	0.56	**< 0.001**
**MRI metrics**					
Evans Index	0.38 (0.05)	0.37 (0.04)	0.40 (0.06)	−0.59	0.2
Callosal Angle	90.63 (22.32)	86.99 (22.52)	99.99 (20.37)	−0.59	0.14
Presence of DESH	53 (53%)	45 (67%)	8 (24%)	0.43	**< 0.001**

^*1*^ Mean (SD); n (%)

^*2*^ Cohen’s D; Standardized Mean Difference

^*3*^ Wilcoxon rank sum test; Pearson’s Chi-squared test

The change in UPDRS scores after CSFTT revealed a notable effect size of −0.96, which was statistically significant (*p* = 0.016). Furthermore, the percentage variation post-procedure in TUG scores had an effect size of 1.1, which was statistically significant (*p* = 0.019).

At baseline, iNPH patients demonstrated significantly higher MMSE scores with an effect size of 0.99 (*p* = 0.031), while FAB scores showed no significant difference between groups (*p* = 0.5). Following CSFTT, the MMSE difference revealed a substantial effect size of 1.26 (*p* = 0.005), and the FAB difference showed an effect size of 0.81 (*p* = 0.019), indicating significant cognitive improvement in the iNPH group. There was no significant difference in the distribution of incontinence between the two groups.

Evaluations of CSF dynamics metrics showed that the P_start_ was not significantly different between the two groups. However, P_plateau_ and R_out_ showed statistically significant differences with effect sizes of 0.89 and 0.56, respectively, with p-values below the 0.001 mark.

EI and CA showed no statistically significant differences between the two groups. However, DESH features showed a statistically significant difference with an effect size of 0.43, with a p-value below the 0.001 mark.

### Volume analysis

Volume analysis did not show any statistically significant difference in the total brain volume, cortical gray matter, subcortical gray matter, white matter, and ventricular volumes between the two groups.

### Cortical thickness analysis

**Fig. 2:**
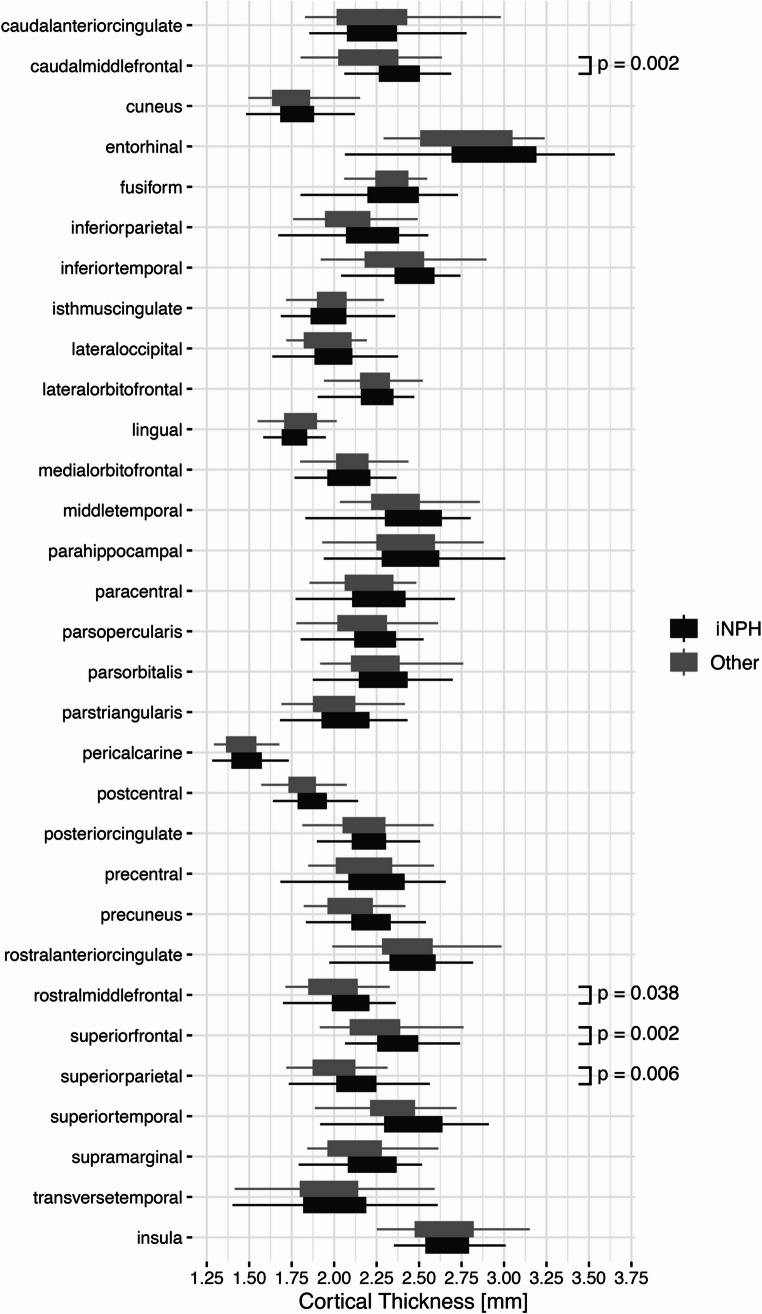
Boxplots illustrating the difference in cortical thickness between iNPH patients and ‘Other’. Statistical significance was set at the level of false discovery rate corrected *p* < 0.05

**Fig. 3:**
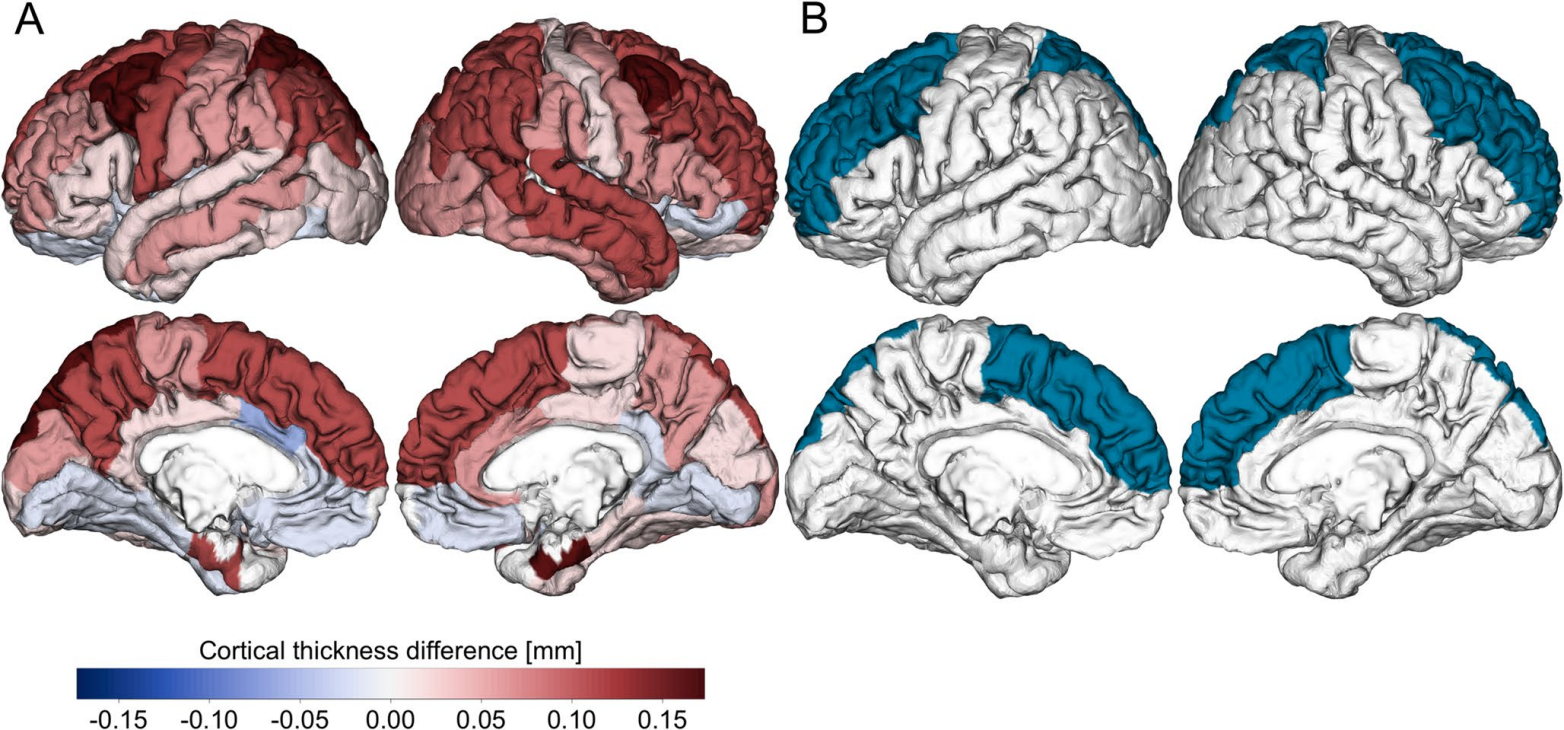
(**A**) Difference in cortical thickness between iNPH patients and ‘Other’; (**B**) Regions of significant difference after correction for multiple comparisons. Statistical significance was set at the level of false discovery rate corrected *p* < 0.05

In the frontal lobe, the caudal middle frontal region demonstrated a significant difference in thickness between the two groups (Cohen’s D = 0.86, corrected *p* = 0.002). The rostral middle frontal and superior frontal areas also showed significant differences (corrected *p* = 0.038 and 0.002, respectively). Additionally, the superior parietal region in the parietal lobe displayed a notable difference with an effect size of 0.80 and a statistically significant corrected p-value of 0.006. The cortical thickness analysis results are reported in Table 2 and shown in Figs. 2 and 3.

**Table 2:** Cortical thickness analysis

Thickness		Group				
	Overall, *N* = 100^*1*^	NPH, *N* = 67^*1*^	Other, *N* = 33^*1*^	Effect Size^2^	*p*-value^3^	Corrected *p*-value^4^
Caudal anterior cingulate	2.23 (0.23)	2.22 (0.21)	2.23 (0.28)	−0.03	0.8	> 0.9
Caudal middle frontal	2.31 (0.20)	2.36 (0.17)	2.20 (0.21)	0.86	< 0.001	**0.002**
Cuneus	1.78 (0.14)	1.80 (0.14)	1.75 (0.14)	0.35	0.13	0.3
Entorhinal	2.89 (0.35)	2.94 (0.37)	2.80 (0.30)	0.38	0.080	0.2
Fusiform	2.34 (0.18)	2.34 (0.19)	2.34 (0.16)	0.03	0.9	> 0.9
Inferior parietal	2.18 (0.20)	2.21 (0.20)	2.10 (0.19)	0.54	0.020	0.11
Inferior temporal	2.42 (0.20)	2.44 (0.18)	2.38 (0.23)	0.33	0.2	0.3
Isthmus cingulate	1.99 (0.15)	1.98 (0.16)	1.99 (0.14)	−0.04	0.9	> 0.9
Lateral occipital	1.99 (0.15)	2.00 (0.16)	1.95 (0.15)	0.31	0.2	0.3
Lateral orbitofrontal	2.25 (0.14)	2.25 (0.14)	2.24 (0.13)	0.07	0.9	> 0.9
Lingual	1.78 (0.10)	1.77 (0.09)	1.79 (0.12)	−0.21	0.2	0.4
Medial orbitofrontal	2.09 (0.15)	2.08 (0.15)	2.10 (0.16)	−0.11	0.4	0.5
Middle temporal	2.42 (0.22)	2.45 (0.22)	2.36 (0.22)	0.41	0.084	0.2
Parahippocampal	2.43 (0.26)	2.43 (0.27)	2.42 (0.24)	0.02	> 0.9	> 0.9
Paracentral	2.25 (0.19)	2.27 (0.20)	2.20 (0.17)	0.38	0.10	0.2
Pars opercularis	2.21 (0.18)	2.23 (0.17)	2.17 (0.20)	0.33	0.2	0.3
Pars orbitalis	2.28 (0.20)	2.28 (0.20)	2.27 (0.22)	0.06	> 0.9	> 0.9
Pars triangularis	2.06 (0.18)	2.08 (0.17)	2.03 (0.18)	0.30	0.2	0.3
Pericalcarine	1.47 (0.12)	1.49 (0.12)	1.45 (0.11)	0.34	0.14	0.3
Postcentral	1.86 (0.13)	1.88 (0.12)	1.82 (0.13)	0.46	0.043	0.2
Posterior cingulate	2.19 (0.15)	2.20 (0.14)	2.18 (0.17)	0.13	0.4	0.5
Precentral	2.23 (0.21)	2.26 (0.20)	2.16 (0.21)	0.47	0.037	0.2
Precuneus	2.17 (0.19)	2.21 (0.19)	2.10 (0.17)	0.61	0.010	0.061
Rostral anterior cingulate	2.45 (0.22)	2.47 (0.21)	2.43 (0.24)	0.16	0.4	0.6
Rostral middle frontal	2.06 (0.16)	2.09 (0.15)	1.99 (0.18)	0.62	0.005	**0.038**
Superior frontal	2.34 (0.18)	2.38 (0.16)	2.24 (0.19)	0.82	< 0.001	**0.002**
Superior parietal	2.09 (0.19)	2.13 (0.18)	1.99 (0.16)	0.80	< 0.001	**0.006**
Superior temporal	2.42 (0.23)	2.45 (0.22)	2.36 (0.22)	0.39	0.088	0.2
Supramarginal	2.18 (0.19)	2.21 (0.18)	2.12 (0.20)	0.46	0.046	0.2
Transverse temporal	1.99 (0.29)	2.00 (0.29)	1.97 (0.28)	0.11	0.6	0.7
Insula	2.66 (0.19)	2.65 (0.17)	2.68 (0.23)	−0.15	0.4	0.6

^*1*^ Mean (SD)

^*2*^ Cohen’s D

^*3*^ ANCOVA with *Age* and *Sex* as covariates

^*4*^ Benjamini & Hochberg correction for multiple testing

### Relationship of cortical thickness and clinical assessments

The superior frontal region and the precuneus manifested significant inverse correlations with the difference in UPDRS scores, with coefficients of −0.324 (*p* = 0.0366) and − 0.313 (*p* = 0.0433), respectively. This suggests that a reduction in cortical thickness in these regions is correlated with a smaller magnitude of change in UPDRS scores, reflecting a lesser degree of symptom relief post-procedure. Similarly, the superior parietal region revealed a near-significant inverse correlation (*R* = −0.302, *p* = 0.0518), hinting at a possible similar trend or pattern.

No significant correlation was found with TUG scores.

### Predictive modeling for normal pressure hydrocephalus

To systematically evaluate the incremental diagnostic value of cortical thickness analysis, we defined 8 distinct predictor configurations:

- Model A – Imaging Only: EI, CA, DESH.
- Model B – Clinical + CSF Dynamics: UPDRS, MMSE, FAB, R_out_.
- Model C – Combined conventional (Model A + Model B).
- Model D – Cortical Thickness only.
- Model E – Cortical Thickness + Imaging (Model A + Model D).
- Model F – Cortical Thickness + R_out_.
- Model G – Cortical Thickness + Clinical.
- Model H – Full combined.

**Fig. 4:**
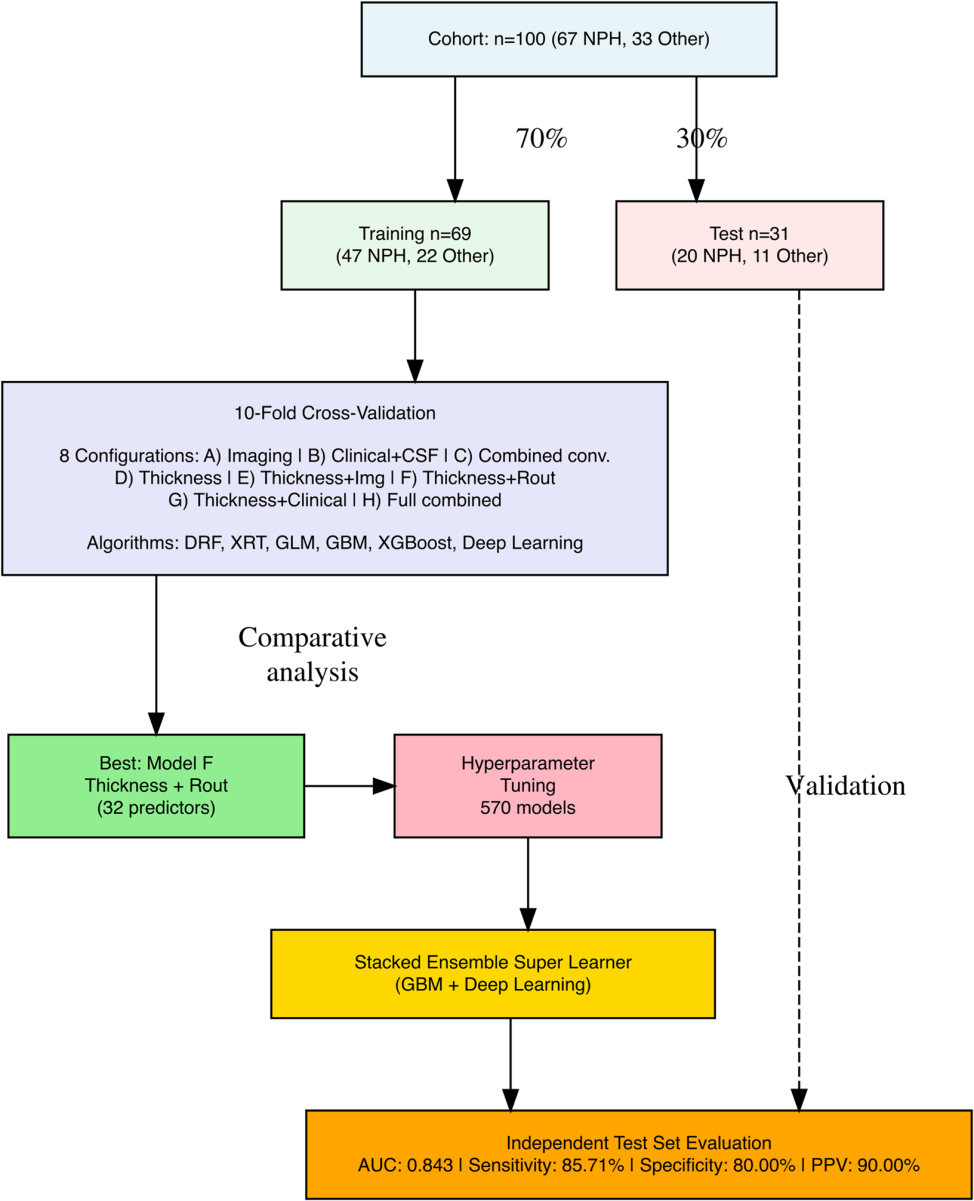
Study design flowchart for machine learning-based prediction of shunt responsiveness in iNPH

The dataset was initially split into a training set (70%) and an independent test set (30%), stratified by outcome. All model selection procedures were performed exclusively on the training set, while the test set remained untouched until the final validation stage (Fig. 4).

For each predictor configuration, multiple machine learning algorithms were trained on the training set, including DRF, XRT, GLM with Regularization, GBM, XGB, and Deep Learning machines. Algorithm comparison and selection were conducted using 10-fold cross-validation within the training set, with the area under the ROC curve (AUC) as the primary performance metric. The cross-validated results for each configuration are reported in Table 3. Based on cross-validated AUC, Model F, combining average cortical thickness measurements (31 ROIs) with the R_out_ value from CSF dynamic evaluation, demonstrated the most favorable performance among the evaluated configurations.

**Table 3:** Cross-validated performance of the eight predictor configurations evaluated on the training set only using 10-fold cross-validation. Reported values represent the mean AUC across folds

Model	Predictor configuration	Algorithm	AUC (10-fold CV, mean ± SD)
A	EI, CA, DESH	GLM	0.679 ± 0.013
B	UPDRS, MMSE, FAB, R_out_	GBM	0.680 ± 0.027
C	EI, CA, DESH, UPDRS, MMSE, FAB, R_out_	Deep Learning	0.724 ± 0.104
D	Cortical thickness (31 ROIs)	Deep Learning	0.673 ± 0.116
E	Cortical thickness (31 ROIs), EI, CA, DESH	GLM	0.693 ± 0.072
**F**	**Cortical thickness (31 ROIs)**,** R**_**out**_	Deep Learning	**0.759 ± 0.078**
G	Cortical thickness (31 ROIs), UPDRS, MMSE, FAB	Deep Learning	0.723 ± 0.094
H	Cortical thickness (31 ROIs), EI, CA, DESH, UPDRS, MMSE, FAB, R_out_	Deep Learning	0.690 ± 0.104

Following the selection of Model F, we performed extensive hyperparameter tuning and algorithmic exploration exclusively within the training set for this configuration, comprising 32 predictors derived from the DKT atlas cortical thickness measures and Rout. A total of 570 candidate models were trained and evaluated using internal cross-validation. From this process, a stacked ensemble Super Learner emerged as the optimal model, integrating predictions from a GBM and a Deep Learning model.

**Fig. 5:**
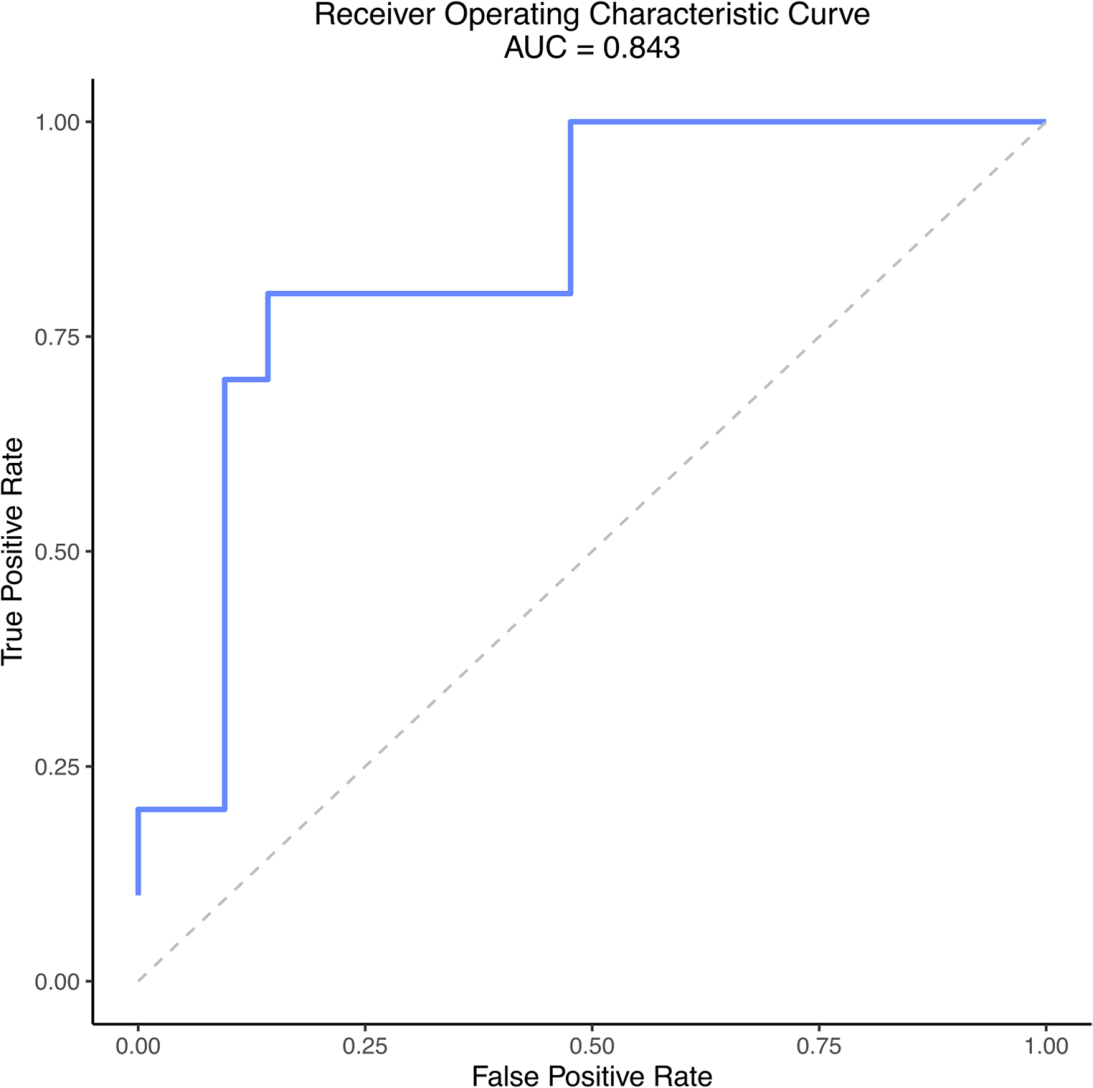
Receiver Operating Characteristic Curve for Super Learner model. AUC: Area Under the Curve

Only after the full model architecture and hyperparameters were finalized, the Super Learner was retrained on the entire training set and evaluated once on the independent test set. As reported in Table 4, the final model achieved a sensitivity of 85.71%, a specificity of 80.00%, and a positive predictive value of 90.00% on the test set. The corresponding ROC curve is shown in Fig. 5, with an AUC of 0.843.

**Table 4:** Confusion matrix for Super Learner on unseen test data

	**Predicted NPH**	**Predicted Other**
**Actual NPH**	18	3
**Actual Other**	2	8
Sensitivity = $\frac{TP}{TP+FN}$ = 85.71%		
Specificity = $\frac{TN}{TP+FN}$ = 80.00%		
Positive Predictive Value = $\frac{TP}{TP+FP}$ = 90.00%		
Negative Predictive Value = $\frac{TN}{TN+FN}$ = 72.73%		
Accuracy = $\frac{TP+TN}{TP+TN+FP+FN}$ = 83.87%		

**Fig. 6:**
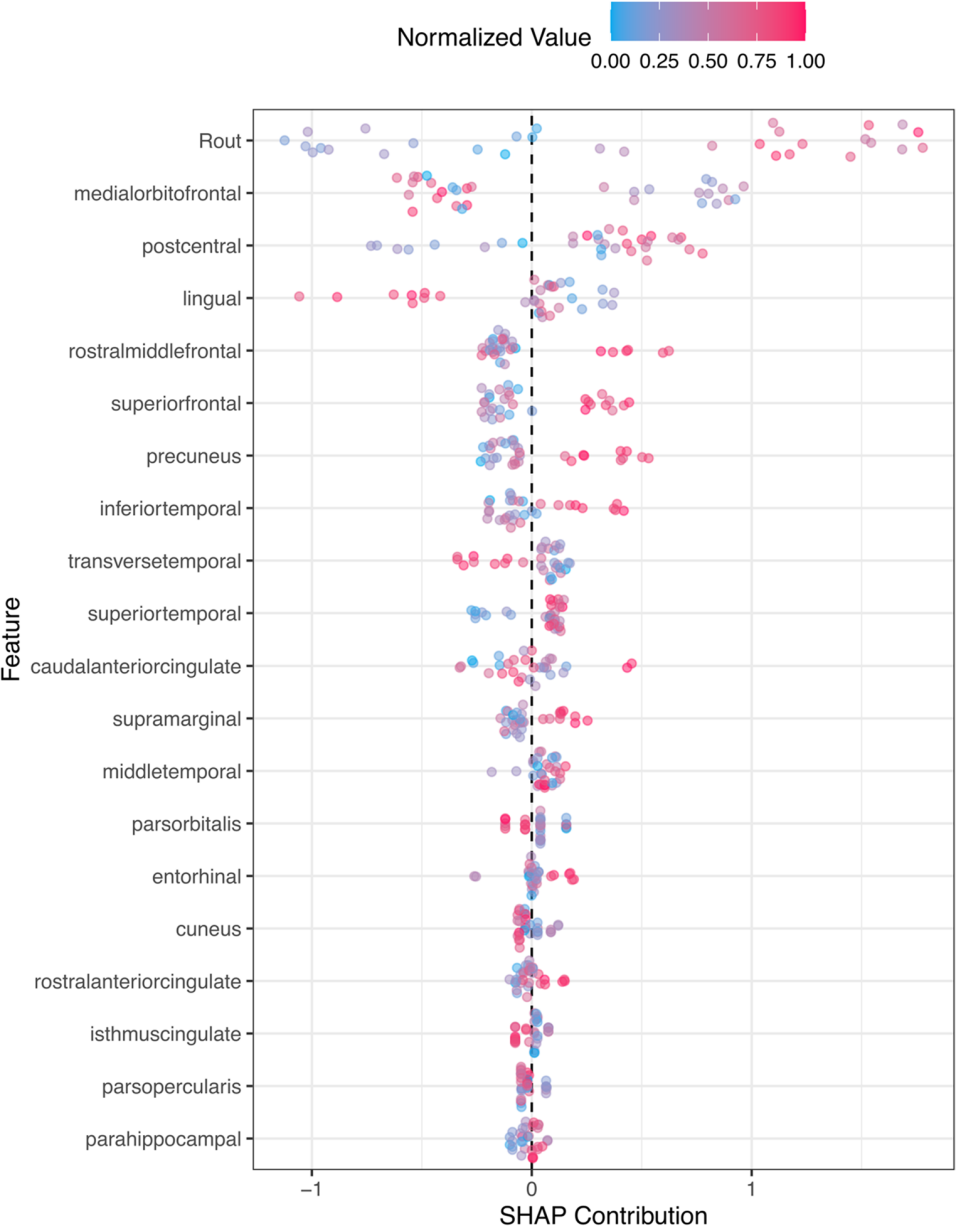
SHAP contribution plot illustrating the relative importance of the most important predictors. SHAP: SHapley Additive exPlanations; Rout: cerebrospinal fluid resistance to outflow

The SHAP Contribution plot, presented in Fig. 6, provides a detailed analysis of the predictors’ influence on the model’s predictions. Notably, the R_out_ variable emerges as a pivotal predictor, affirming its well-established significance in iNPH. Furthermore, regions within the frontal and parietal areas rank prominently, underscoring their determinant roles in the model’s decision process.

## Discussion

Idiopathic normal-pressure hydrocephalus stands as a unique and distinct form of progressive dementia that is both primitive and, to an extent, reversible. VPS implantation has been found to alleviate symptoms, with a success rate ranging between 33% and 84%. Such outcome variations are influenced by factors such as the timing of the intervention, methodologies in outcome assessments, and criteria for patient selection [3, 41–43, 44]. Diagnosing iNPH relies on a confluence of clinical presentations, neuroradiological findings, and specific invasive diagnostic procedures [3]. Among the clinical manifestations, gait disturbances are almost universally observed, presenting in 94 to 100% of iNPH patients. Concurrently, cognitive impairments and urinary dysfunctions are noted with varying frequencies, affecting between 78 and 98% and 60–92% of patients, respectively. Interestingly, the simultaneous presence of all these symptoms is observed in approximately 60% of the diagnosed iNPH population [3].

Invasive diagnostic procedures, including CSFTT, help differentiate iNPH from other forms of dementia. The tap test has a sensitivity of 58% and a specificity of 75%, while elevated R_out_ values have shown a PPV of over 80% for VPS success [3].

Cortical thickness measurements, captured across varied brain regions, have recently gained prominence in studying normal development and aging and in the context of neurodegenerative diseases [27, 28, 30–33, 34]. In the context of iNPH, a study by Kang et al. in 2013 contrasted cortical thickness between CSFTT responders and non-responders, juxtaposing these findings with an AD cohort. The results hinted at potential comorbid AD in TT non-responders, suggesting that coexisting AD might influence this subgroup’s observed cortical thinning patterns [27]. A subsequent study by the same group showed significant cortical thickening in frontal, parietal, and occipital regions, as well as cortical thinning in temporal and orbitofrontal areas in iNPH patients compared to healthy controls [28]. Another study by Lang et al. showed decreased GM volume in the right supplementary motor area and in the right posterior parietal cortex for CSFTT non-responders [29]. A study by Belgrado et al. evaluated extrapyramidal signs in the diagnosis of iNPH, showing significant differences in MDS-UPDRS-III scores after CSFTT for definitive and probable iNPH compared to not-iNPH [45].

Our findings align with prior research, drawing attention to notable disparities in cortical thickness, particularly in the middle frontal, superior frontal, and superior parietal regions. Drawing parallels with previously established links between cortical thickness and gait disturbances, our results emphasize the value of assessing regional cortical thickness as a pivotal diagnostic and prognostic indicator for iNPH. Notably, the superior frontal and precuneus regions presented significant inverse correlations with the variations in UPDRS scores post-CSF subtraction. Furthermore, the subtle trend observed in the superior parietal region, while just shy of statistical significance, aligns with these findings. The consistent pattern across these regions suggests that certain areas of the brain, when manifesting reduced cortical thickness, may indicate a less favorable response to interventions like CSF subtraction.

Our study demonstrates the effectiveness of the Super Learner stacked ensemble model in predicting iNPH by leveraging multiple ML techniques. The model’s use of predictors from the frontal and parietal regions aligns with previous research and reinforces their potential relevance in diagnosis. Moreover, the success of our predictive model becomes even more pronounced when considering the PPV of 90% achieved when considering both R_out_ and cortical thickness. This represents a significant enhancement from the approximate 80% PPV when relying on R_out_ alone, underscoring the value of incorporating multiple parameters in iNPH diagnosis. This comprehensive approach, informed by fluid dynamics and structural neuroimaging, may pave the way for more nuanced and tailored therapeutic interventions.

## Limitations

Although this study provides novel insights into the diagnostic value of cortical thickness analysis in iNPH, several limitations should be acknowledged. First, the relatively small sample size, while comparable to prior single-center iNPH studies, may limit the generalizability of the findings and increase variability in model performance estimates. To mitigate this, we employed strict training–testing separation and cross-validation–based model selection; however, larger datasets would enable more stable estimation of model parameters and further refinement of complex models, such as ensemble learners.

Second, the study was conducted at a single tertiary referral center, which may introduce center-specific biases in patient selection, imaging protocols, or clinical evaluation. Although standardized diagnostic criteria and imaging procedures were applied, external validation in independent cohorts from other institutions is necessary to confirm the robustness and transportability of the proposed model.

Third, although internal cross-validation was used for model selection and hyperparameter tuning, an independent external validation cohort was unavailable. Although the final model was evaluated on a strictly held-out test set, future studies should aim to validate the proposed approach across multicenter datasets and heterogeneous imaging platforms to further assess real-world performance.

Finally, cortical thickness measurements were derived from automated segmentation pipelines. Although extensively validated, these pipelines may be sensitive to image quality and scanner-specific characteristics. Future work should explore harmonization strategies and assess the impact of acquisition variability on model performance.

## Conclusions

Our analysis suggests that patients who exhibit negative CSFTT or remain unresponsive to VPS may have a distinct cortical thinning pattern. Such patterns may indicate comorbidities or alternative neurological pathologies. Consequently, a comprehensive and multi-dimensional diagnostic approach is necessary for iNPH to address overlapping conditions.

## Data Availability

The datasets generated and analysed during the current study are available from the corresponding author on reasonable request.

## Abbreviations

AD: Alzheimer’s disease
AI: artificial intelligence
ANCOVA: analysis of covariance
AUC: area under the curve
CSF: cerebrospinal fluid
CSFTT: cerebrospinal fluid tap test
DARTEL: diffeomorphic anatomic registration through exponentiated lie
DESH: disproportionately enlarged subarachnoid–space hydrocephalus
DKT: Desikan–Killiany–Tourville
DRF: distributed random forest
FLAIR: fluid–attenuated inversion recovery
GBM: gradient boosting machine
GLM: generalized linear model
GM: gray matter
iNPH: idiopathic normal pressure hydrocephalus
ML: machine learning
MRI: magnetic resonance imaging
PPV: positive predictive value
PS: Parkinson’s spectrum
RF: random forest
ROC: receiver operating characteristic
SD: standard deviations
SHAP: Shapley additive explanations
SVM: support vector machine
TUG: timed up and go test
UPDRS: unified Parkinson’s disease rating scale
VaD: vascular dementia
VPS: ventriculoperitoneal shunt
WM: white matter
XGB: extreme gradient boosting machine
XRT: extremely randomized trees
